# The anti-fibrotic effect of GV1001 combined with gemcitabine on treatment of pancreatic ductal adenocarcinoma

**DOI:** 10.18632/oncotarget.12057

**Published:** 2016-09-16

**Authors:** Joo Kyung Park, Yejin Kim, Hyemin Kim, Jane Jeon, Tae Wan Kim, Ji-Hong Park, Young-il Hwnag, Wang Jae Lee, Jae Seung Kang

**Affiliations:** ^1^ Division of Gastroenterology, Department of Medicine, Samsung Medical Center, Sungkyunkwan University School of Medicine, Seoul, Korea; ^2^ Laboratory of Vitamin C and Anti-Oxidant Immunology, Department of Anatomy and Cell Biology, Seoul National University College of Medicine, Seoul, Korea; ^3^ Institute of Allergy and Clinical Immunology, Seoul National University Medical Research Center, Seoul, Korea; ^4^ Department of Ophthalmology, Seoul Metropolitan Government-Seoul National University Boramae Medical Center, Seoul, Korea; ^5^ Department of Rehabilitation Medicine, Seoul National University Bundang Hospital, Bundang-gu, Seongnam-si, Gyeonggi-do, Korea

**Keywords:** GV 1001, gemcitabine, xenograft tumor model, pancreatic ductal adenocarcinoma

## Abstract

GV1001 is a telomerase-based cancer vaccine made of a 16-mer telomerase reverse transcriptase (TERT) peptide, and human TERT, the rate-limiting subunit of the telomerase complex, is an attractive target for cancer vaccination. The aim of this study was to evaluate the effect of telomerase peptide vaccination, GV1001 combined with gemcitabine in treatment of pancreatic ductal adenocarcinoma (PDAC). Human PDAC cell lines were used *in vitro* experiment and also, PDAC xenograft mice model was established using PANC1, AsPC1 and CD133+ AsPC1 (PDAC stem cell). Treatment groups were divided as follows; control, gemcitabine, GV1001, gemcitabine and GV1001 combination. The inflammatory cytokines were measured from the blood, and xenograft tumor specimens were evaluated. GV1001 treatment alone did not affect the proliferation or the apoptosis of PDAC cells. Gemcitabine alone and gemcitabine with GV1001 groups had significantly reduced in tumor size and showed abundant apoptosis compared to other treatment groups. Surprisingly, xenograft PDAC tumor specimens of gemcitabine alone group had been replaced by severe fibrosis whereas gemcitabine with GV1001 group had significantly less fibrosis. Blood levels of tumor necrosis factor (TNF)-α, interleukin (IL)-6 and IL-1β increased in gemcitabine alone group, however, it was decreased in gemcitabine with GV1001 group. GV1001 combined with gemcitabine treatment showed significant loss of fibrosis in tumor tissue as well as tumor cell death. Therefore, further investigation of GV1001 effect combined with gemcitabine treatment may give us useful insights to overcome the hurdle in anti-cancer drug delivery over massive fibrosis around PDACs.

## INTRODUCTION

Pancreatic ductal adenocarcinoma (PDAC) is a very aggressive human cancer and has dismal prognosis with only 6% of patients survive 5 years after diagnosis [1–4]. In spite of the progresses of treatments, the attempts at improving survival of patients with PDAC in the past 15 years, especially in the advanced disease setting, have failed and resulted in no significant improvement [5]. Surgical resection is the only potentially curative treatment and only 15% of patients could be candidate for resection [6, 7]. Some chemotherapeutic agents have been used in treatment of PDACs, and gemcitabine became the standard chemotherapeutic agent in pancreatic cancer after randomized trial in 1997 [8]. Gemcitabine is a nucleoside pyrimidine analogue which exerts its cytotoxic actions primarily by the incorporation of gemcitabine triphosphate into DNA, leading to masked chain termination [9]. However, pancreatic cancer is highly resistant to chemotherapy including gemcitabine, and the most disappointing circumstance is mainly due to the late diagnosis of PDAC [10, 11]. In addition, the best supportive care and maintain the better quality of life are critical since the majority of the patients with PDACs are in advanced stage.

Some unique characteristics of PDACs such as high stromal-to-epithelial ratio (desmoplasia), restricted vasculature and hypoxic environment, may disturb the drug delivery for chemotherapy to the tumor thereby explaining the limited benefits observed to-date [12, 13]. The high proportion of stromal cells in PDAC is associated with overexpression of several growth factors and cytokines, which causes resistance to anti-cancer drug and is also related to poor chemotherapy response rate or patient prognosis. Drug delivery to pancreatic tumors is especially difficult because it has hypovascular and poorly perfused nature. The presence of stomal components increases the interstitial fluid pressure and prevents drugs from penetrating the tissue interstitium [12, 14]. To date, many efforts to find an appropriate combination of multi-therapeutic agents with different modes of action to overcome the chemoresistance have been made during the past years. The most of these chemotherapeutic agents have not been successful enough to show the significant survival benefit [15–21]. Thus, more effective treatment strategies are highly required, and immunotherapy for target which is critical in cancer growth seemed to be a promising approach [22, 23].

In the process of repeated rounds of DNA replication, the telomeric ends of DNA become progressively shortened and without a compensatory mechanism cells senesce and die [24, 25]. Therefore, telomerase expression is essential for the proliferation of most cancer cells, but the enzyme is inactive in the majority of normal human tissues. Reactivation of telomerase, the telomere-repair enzyme, is a crucial event in oncogenic transformation, and is highly expressed in essentially all cancer forms, while the expression in normal tissues is restricted [26]. Moreover, telomerase activity is considered indispensable for tumor immortalization and growth and occurs in nearly all pancreatic cancers [26]. That means that inhibiting the chromosome-elongating enzyme should, in theory, be a relatively safe and effective way to, if not directly kill, at least weaken cancer cells before treating with other agents. Therefore, human telomerase reverse transcriptase (hTERT), the rate-limiting subunit of the telomerase complex, is an attractive target for cancer vaccination.

GV1001 is a human telomerase reverse transcriptase catalytic subunit (hTERT) class II 16 mer peptide vaccine [24, 25]. Bernhardt et al reported in their phase 2 trials that GV1001 treatment in advanced pancreatic cancer showed a total immune response in 24 (63%) of 38 patients and those responders had a greater median survival (216 days) than did non-responders (88 days) [27]. Although, cytotoxic drugs are generally regarded as immunosuppressive, some chemotherapy regimens might potentiate the effect of cancer vaccines [28–33]. The preclinical data clearly showed immunogenicity of GV1001 in patients with PDACs that the synergy of gemcitabine with cancer vaccines and the other positive immunomodulatory effects of gemcitabine and fluoropyrimidines [26]. Therefore, the aims of this study were to investigate the effect of telomerase peptide vaccination, GV1001 combined with gemcitabine in treatment of PDAC.

## RESULTS

### Direct cytotoxic effect of GV1001 in PDAC cells

**Figure 1:**
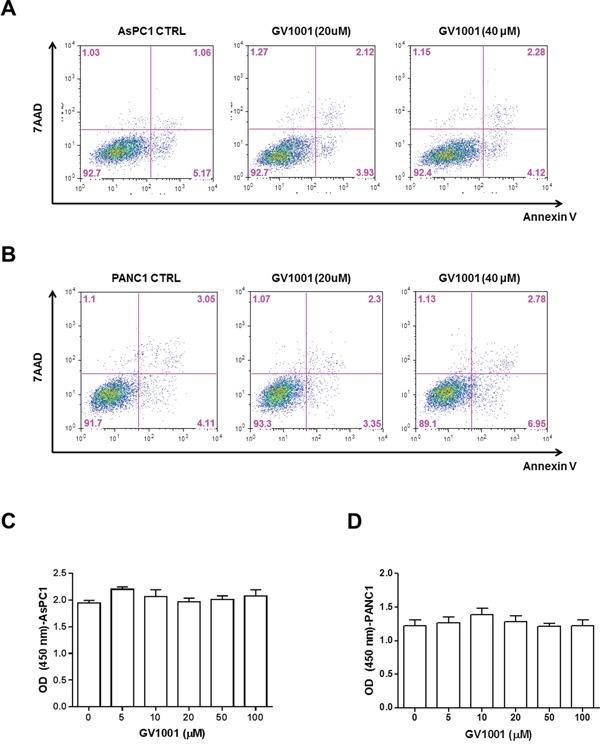
Direct cytotoxic effect of GV1001 in PDAC cell lines PDAC cells were treated with GV1001 (20 and 40 μM) for 24 hrs. **A.** AsPC1 or **B.** PANC1 cells were stained with Annexin V and 7-AAD, and analyzed by flow cytometry. And, the proliferation and viability of **C.** AsPC1 and **D.** PANC1 cells were examined by CCK-8 assay (n=4).

Direct cytotoxic effect of GV1001 was evaluated in PDAC cells. Flow cytometry analysis was done treated with GV 1001 for 24 hrs. In Figure 1A and 1B, PDAC cell lines, PANC1 and AsPC1, were tested according to the different concentration of GV1001. There were 3 groups according to the different concentration of GV1001 treatment (control, 20 μM and 40 μM). Figure 1A showed 6.23%, 6.03% and 6.4% of Annexin V-positive apoptotic cells in each group, and Figure 1B showed 7.16%, 5.65% and 9.73%: control, GV1001 20 μM and GV1001 40 μM accordingly. In addition, the number of cells in early stages of apoptosis and the late stage of apoptosis didn't make any significant statistical differences compared to the control group and also according to the concentration of GV1001. In addition, PDAC cells were treated with GV1001 in various concentrations for 24 hrs, and viability and proliferation were accessed by CCK-8 assay (Figure 1C and 1D). AsPC1 and PANC1 cells were not affected their viabilities via GV100 treatment. Therefore, we could observe that GV1001 did not have direct cytotoxic effect to PDAC cells.

### Changes in body weight and tumor volume upon GV1001 and gemcitabine treatments in PDAC xenograft mice

**Figure 2:**
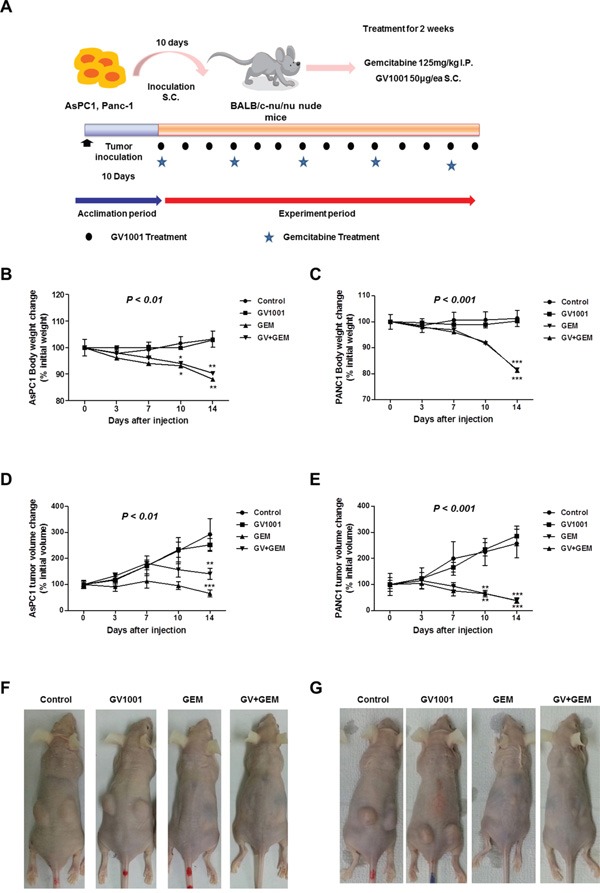
Changes of body weight and tumor volume upon GV1001 and gemcitabine treatments **A.** An experimental scheme of PDAC xenograft model. AsPC1 or PANC1 cells (1×10^6^/mice) were inoculated to the 4 different groups of BALB/c nude mice (control, GV1001, GV1001+gemcitabine and gemcitabine alone). After 10 days of inoculation, gemcitabine (125 mg/kg) was intraperitoneally (i.p.) injected twice a week, and GV1001 (50 μg/mice) was subcutaneously (s.c.) injected once a day. GV1001 and gemcitabine were injected for 2 weeks, and mice were sacrificed for further experiments. The body weight **B** and **C.** and tumor volume **D** and **E.** were measured twice a week for AsPC1 (B and D) and PANC1 (C and E) xenograft mice, which were represented as percentage of each initial value. The representative picture of xenograft PDAC model among different treatment groups; **F.** AsPC1 xenograft mice **G.** PANC1 xenograft mice.

Figure 2A showed an experimental scheme for PDAC xenograft model and drug treatments. There were 4 different treatment groups: control, GV1001 alone, gemcitabine alone and GV1001+gemcitabine. After the completion of protocol (10 days of tumor growth time + 14 days of treatment period), each group of mouse was sacrificed and the gross tumor growth as well as any signs of PDAC cell dissemination were examined. PDAC xenograft tumor was successfully established and all of the study mice had a pair of ovoid PDAC tumors on both sides of buttocks. In addition, there was no treatment related mortalities. In the xenograft mouse model of AsPC1 and PANC1, the body weight of mice was checked twice a week and the changes of their weight were represented in Figure 2B and 2C. The body weight of mice in control and GV1001 alone treatment groups didn't change much during the study. However, mice in gemcitabine alone and GV1001+gemcitabine treatment groups had sudden decrement of body weight along with the chemotherapeutic agents. Interestingly, body weight of GV1001 combination with gemcitabine group was not dramatically significant compared to gemcitabine alone treatment group. PDAC xenograft tumors had different tumor size at the end of treatment, and there were clearly demarcated tumors in control and GV1001 alone treatment groups. On the other hands, tumors in GV1001+gemcitabine and gemcitabine alone groups were nearly disappeared or significantly shrunk in the gross examination of study mice. The length and width of each tumor were measured, and volume was calculated using above mentioned formula: tumor volume = (length x width^2^) x π/6 [34]. PDAC xenograft tumors of AsPC1 and PANC1 in the same treatment groups did not show significant difference in terms of tumor volume. However, there was statistically significant volume decrease in gemcitabine alone and gemcitabine+GV1001 treatment groups from PDAC xenograft tumors of both AsPC1 and PANC1 (Figure 2D-2F). In terms of GV1001 effects on PDAC xenograft tumors, GV1001 single treatment did not show anti-cancer effect compared to the control group. Whereas gemcitabine alone and GV1001+gemcitabine treatment groups had significant decrement of tumor size and they didn't show the significant difference in terms of decreased tumor size.

### Reduced fibrosis after the treatment of GV1001 combined with gemcitabine

**Figure 3:**
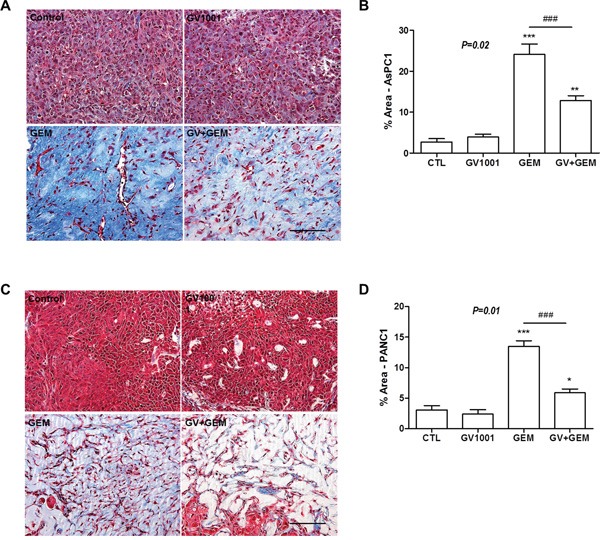
Changes of fibrosis in xenograft PDAC tumor among the different treatment groups After GV1001 and gemcitabine injection for 2 weeks, **A, B.** AsPC1 and **C, D.** PANC1 tumors were excised and fixed. Tumor tissues were embedded in paraffin, sectioned with 5 μm thickness and stained with Masson's Trichrome; Scale bar, 100 μm. (A, C) Masson's trichrome staining for xenograft PDAC tissue after the different treatment. (B, D) The area occupied by blue-stained collagen was quantified using the ImageJ program and we could observe the significantly more fibrosis in Gemcitabine group compared to Gemcitabine +GV1001 group.

After the harvesting PDNA xenograft tumors, Masson's trichrome staining was done for each mice; PDAC AsPC1 xenograft model (Figure 3A). Gemcitabine alone and gemcitabine+GV1001 groups had significant amount of reduced tumor tissue and it was confirmed in pathologic specimens as well. Although both treatment groups which containing gemcitabine did not show significant difference in tumor size, there was marked difference in terms of fibrosis in pathologic specimens between the two groups. Gemcitabine alone treatment group showed the abundant fibrosis replaced the tumor tissue whereas gemcitabine+GV1001 treatment group had significant reduced fibrosis compared to gemcitabine alone treatment group, however, both groups had significant tumor cell death.

### PDAC stem cells and establishing xenograft tumor model

**Figure 4:**
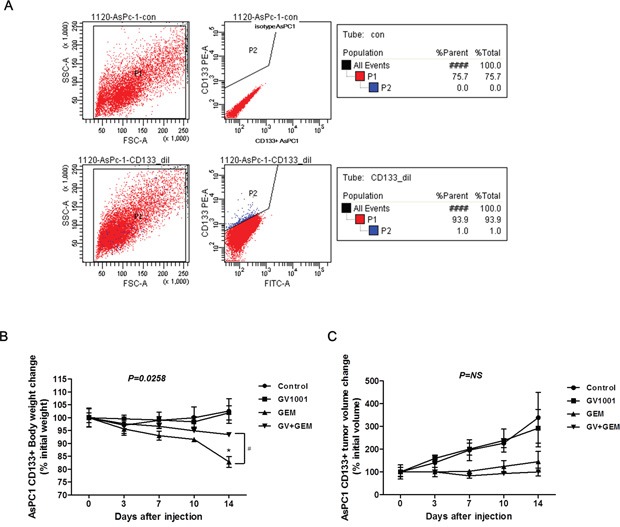
Changes in CD133+ AsPC1 xenograft mice after GV1001 and gemcitabine treatments **A.** AsPC1 cells were stained with isotype control (upper panel) or anti-CD133 antibody (lower panel), and CD133+ AsPC1 cells were examined by flow cytometry. CD133+ AsPC1 cells were sorted out, and isolated cells (1×10^6^/mice) were inoculated to BALB/c nude mice. After xenograft tumors were firmly established, gemcitabine (125 mg/kg) was injected (i.p.) twice a week, and GV1001 (50 μg/mice) was injected (s.c.) daily for 2 weeks. **B.** Body weight and **C.** Tumor volume after GV1001 and gemcitabine treatments in CD133+ AsPC1 xenograft mice were followed twice a week, and represented as percent changes to initial average.

We have been studying PDAC stem cell markers such as CD133 and CD24 and tested for various PDAC cell lines as well (Figure 4A & Supplementary Figure S1). In this study, we have used AsPC1 PDAC cell lines and CD133+ AsPC1 cells were approximately 1 % of total AsPC1 cells (Figure 4A). In addition, other studies have been reported that PDAC stem cells are thought to make up 1-5% of pancreatic tumor cells [5]. Kure et al. examined the expression of cancer stem cell markers (CD24, CD44, CD133, CXCR4, ESA, Nestin) in pancreatic intraepithelial neoplasia (PanIN) and PDAC by using immunohistochemistry (IHC) or flow cytometry (FCM) [36]. In the report, proportion of CD133-positive cells in PDAC was 0.54±0.54%. Also, CD133-expressing cells in PDAC cell lines such as PANC1, MIA PaCa2 and KLM1 were 1.61%, 0% and 0%, respectively [36].

### GV1001 effect on xenograft tumor derived from PDAC stem cells

We also investigated the effect of GV1001 in pancreatic cancer stem cells. In PDAC stem cell xenograft tumor model, AsPC1 CD133+mice, the body weight was rapid and significantly decreased in gemcitabine single treatment whereas GV1001+gemcitabine group was not; Figure 4B, p=0.0258. Also, there were no statistically significant tumor volume changes among the different treatment groups (Figure 4C). However, there was a definite trend that gemcitabine and GV1001+Gemcitabine groups had tumor volume loss while control and GV1001 groups have gradual increase of their tumors.

**Figure 5:**
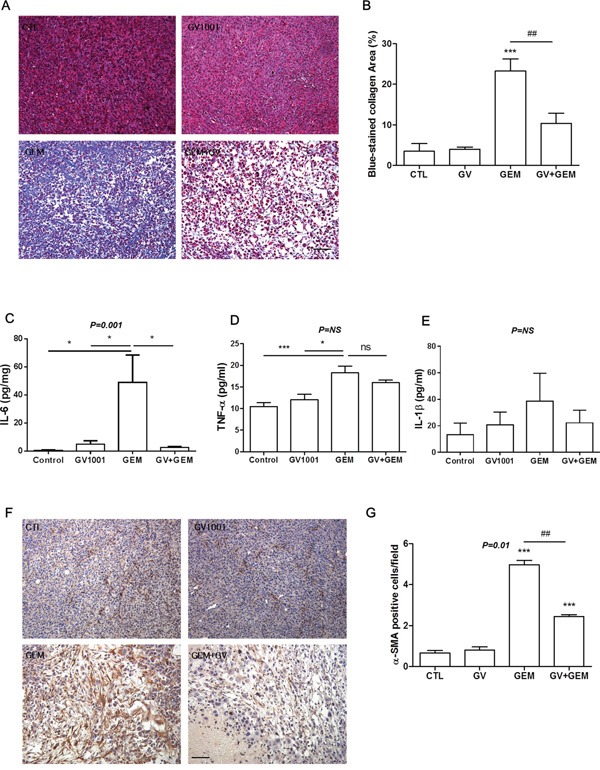
Changes of fibrosis in CD133+ AsPC1 xenograft mice **A.** Paraffin-embedded CD133+ AcPC1 tumor tissues were sectioned and stained with Masson's Trichrome; Scale bar, 100 μm. **B.** Blue-stained area of collagen was quantified by ImageJ software. After GV1001 and gemcitabine treatments for 2 weeks, blood was collected from intra-orbital plexus of each mouse with heparinized capillary. The plasma concentrations of **C.** IL-6, **D.** TNF-α, and **E.** IL-1β were measured by ELISA according to the instructions. **F.** Tumor tissues in CD133+ AcPC1 xenograft mice were immunohistochemically stained with anti-α-SMA antibody (brown color), and nuclei were counterstained with hematoxylin (purple color). Scale bar, 100 μm. **G.** The number of α-SMA immunoreactive cells was quantified by a pathologist described in Materials and Methods.

Followed by Masson's Trichrome staining, intense fibrosis was observed in the tumors of gemcitabine-treated mice, and additional GV1001 treatment reduced the fibrosis (Figure 5A and 5B). At the end of treatment, blood sample of each study mouse was acquired and analyzed for the fibrosis-related cytokines; IL-6, TNF-α and IL-1β (Figure 5C-5E). The levels of TNF-α and especially IL-6 were significantly increased in gemcitabine alone treatment group. The concentration of IL-6 was considerably decreased by GV1001 in a combination with gemcitabine (Figure 5C). TNF-α level also tended to decline by gemcitabine and GV1001 combination (Figure 5D). Although the concentration of IL-1β did not show statistically significant differences among the treatment groups, tendency towards increase or decrease by gemcitabine and GV1001 treatments was observed like IL-6 and TNF-α (Figure 5E). Furthermore, it has been reported that pancreatic stellate cells are the principal source of fibrosis in the stroma and interact closely with cancer cells to create a tumor favorable environment that stimulates local tumor growth and distant metastasis. Alpha smooth muscle actin (α-SMA) is the cytoskeletal protein marker for pancreatic stellate cell activation [35]. Therefore, we examined stellate cells marker, α-SMA in CD133+ AsPC1 xenograft tumor tissues after the treatment (Figure 5F-5G). There was a large amount of stellate cells highly expressing α-SMA were observed in gemcitabine alone group, and it was remarkably decreased in gemcitabine+GV1001 group (Figure 5G).

## DISCUSSION

GV1001 is a telomerase-based cancer vaccine made of a 16-mer TERT peptide, an attractive target for cancer vaccination and the main purpose of this study was to explore the effect of GV1001 when it was combined with gemcitabine in the treatment of PDAC. There were several valuable findings from this study which I would like to address and they are the followings.

First of all, it was observed that GV1001 did not have direct effects on the proliferation nor the apoptosis of PDAC cells *in vitro* experiments and we could say that GV1001 did not show direct anti-cancer effects (Figure 1). It can be explained that GV1001, telomerase peptide vaccine whose mechanism was known to activate combined CD4/CD8 T cell response and it would depend on antigen-presenting cells (APC) [27]. Therefore, it did not show any direct anti-cancer effect *in vitro* experiment. On the other hands, PDAC xenograft mice model showed that treatment groups with gemcitabine alone and gemcitabine combined with GV1001 had significant tumor reduction compared to other groups (Figure 2D and 2E). Although gemcitabine alone or gemcitabine with GV1001 treatment groups had significantly decreased tumor size and volume, there was no significant difference between the two groups. It seemed that anti-cancer effect came from gemcitabine since GV1001 alone treatment group did not have significant reduction in tumor size. In addition, we have created the PDAC stem cell xenograft tumor model with CD133+ AsPC1 cell line (Figure 4). PDAC stem cells are known to be highly chemo-resistant and responsible for early recurrence and metastasis [36, 37]. We could also find out that CD133+ AsPC1 xenograft tumor treated with gemcitabine alone and gemcitabine combined with GV1001 had significant amount of reduced tumor size and abundant apoptosis from the evaluation of xenograft tumor specimens after the sacrifice. Moreover, xenograft PDAC models from AsPC1 and CD133+AsPC1 PDAC cells had significant body weight loss in gemcitabine single treated group compared to gemcitabine+GV1001 treatment group (Figure 4B). Also, the group of mice treated with gemcitabine only became very cachexic and their activities became significantly low compared to gemcitabine+GV1001 treatment group. Those observations lead us to measure the concentration of ghrelin, a hunger hormone, in the blood of each group of mice. Its level was lower in gemcitabine-treated mice, and GV1001 combination increased the level of ghrelin. However, Ghrelin level difference between Gemcitabine only group vs. gemcitabine+GV1001 group was not statistically significant. This result was provided in Supplementary Figure S2; data not shown in result section. With relevance to cachexia, the concentration of Ghrelin, a hunger hormone, was measured in the blood of each group of mice. Although it was not statistically significant among the groups, there was a tendency of increment in serum level of ghrelin in GV1001 containing treatment groups. It seems that the significance of body weight change between gemcitabine only group and gemcitabine+GV1001 group is related with the anti-cachexic effect of GV1001. However, the precise mechanism should be further investigated.

The most interesting finding in this study was GV1001 effect on stroma of PDACs and its microenvironment. Both treatment groups, gemcitabine alone and gemcitabine combined with GV1001, had significant reduction in tumor size, and abundant apoptosis were observed from the xenograft tumor specimens after the sacrifice. Although both treatment groups had significant tumor cell death, tumor specimens of gemcitabine alone treatment had severe fibrosis whereas gemcitabine combined with GV1001 treatment showed significant loss of fibrosis (Figures 3 and 4). Therefore, above observations lead us to study further with the mechanism of GV1001 affecting fibrosis. As we all know, one of the most difficult obstacles which preventing treatment success of PDACs is an early metastasis with rapidly progressive nature, but other immunological and stromal factors are as important as to be overcome [38, 39]. Since chemotherapeutic agents are often administered systemically, drug delivery to solid tumors consists of several processes, including transport via blood vessels, transport across the vessel wall into surrounding tissue, and transport through interstitial space [40]. Among solid tumors, drug delivery to pancreatic tumors is especially difficult because the network of tumor stroma and extracellular matrix (ECM) proteins imposes a barrier for drug delivery. A dense stromal reaction has been shown to impede the penetration of chemotherapeutic agent into PDACs, thus restricting the synergistic potential of chemotherapy [41]. The fibroblasts and fibrotic stroma in pancreatic tumors inhibit the formation and the function of blood vasculature. The spare vasculature is only partially functional and physically separated from the cancer cells by stroma. This unique microenvironment diminishes the drug delivery via blood perfusion and therefore reduces the effectiveness of systemic chemotherapy that relies on functional vasculature for delivery to tumor cells [12]. Several studies were reported that the improvement of vasculature or the depletion of stroma enhanced drug delivery in PDACs. The delivery and efficacy of gemcitabine in mouse pancreatic model was improved by co-administration of a drug that depletes tumor-associated stromal tissue by inhibition of the Hedgehog cellular signaling pathway [42]. The combination of an agonist CD40 antibody with gemcitabine chemotherapy showed tumor regression in both PDAC patient and genetically engineered PDAC mouse model. CD40-activated macrophages rapidly infiltrated tumors, became tumoricidal and facilitated the depletion of stroma [39]. Vitamin D receptor (VDR) is expressed in stroma from human pancreatic tumors and that treatment with the VDR ligand calcipotriol markedly reduced markers of inflammation and fibrosis in pancreatitis and human tumor stroma. VDR acts as a master transcriptional regulator of PSCs to reprise the quiescent state, resulting in induced stromal remodeling, increased intratumoral gemcitabine, reduced tumor volume, and a 57% increase in survival compared to chemotherapy alone [43]. Freig *et al* reported in a trial of an anti-programmed death ligand 1 (PD-L1) antibody that objective responses were reported in malignant melanoma, renal-cell cancer, non-small cell lung cancer (NSCLC) and ovarian cancer, but there were no responders among the 14 patients with advanced PDACs [38, 44]. The relatively poor response in immunotherapy efficacy of PDACs might be related to specific carcinoma-associated fibroblasts (expressing fibroblast activation protein), which secrete C-X-C motif ligand 12 (CXCL12) and thus stop T cells from accessing cancer cell regions in the stroma [44, 45]. In a genetically engineered mouse model of PDAC blocking the receptor of CXCL12, induced rapid T cell accumulation and synergized with anti-PD-L1 in cancer cell killing [44]. Above observations supported that PDAC xenograft tumors responded to GV1001 by reducing tumor fibrosis when they were treated with gemcitabine.

Having massive fibrosis reactions in treatment of gemcitabine alone group from the xenograft PDAC tumors, we have measured blood level of IL-6, TNF-α and IL-1β. Surprisingly, these cytokine levels were elevated in gemcitabine alone treatment group, whereas, they were decreased in gemcitabine with GV1001 combination group (Figure 4D-4F). Moreover, this study showed novel effect of GV1001 which was never been reported in PDAC studies. The mechanism responsible for the fibrosis development in pancreatic cancer has not yet been fully elucidated. In recent years, the activation of pancreatic stellate cells plays a critical role in the pancreatic fibrosis, and pro-inflammatory cytokines such as TNF-α, IL-1, and IL-6 were considered as important candidates for stellate cell activation [46–48]. Especially, IL-6 is assumed to participate in pancreatic fibrosis by activating PSCs and regulate PSC-induced EMT and alterations in gene expression in pancreatic cancer cells [49]. On the other hands, GV1001 was subsequently revealed to show anti-inflammatory effects. In a mouse ischemia-reperfusion injury (IRI) model, this peptide, GV1001 efficiently inhibited the production of IL-6 and MCP-1, which are associated with a decrease in the infiltration of neutrophils and macrophages in the kidney after IRI [50]. Recently, our group reported that GV1001 decreased the production of TNF-α, IL-1β, and IL-6 in peripheral blood mononuclear cells from rheumatoid arthritis patients through the suppression of p38 MAPK and NF-κB activation [51]. Taken together, the increased production of pro-inflammatory cytokines such as TNF-α, IL-1β, and IL-6 in gemcitabine-treated PDAC xenograft mice was suppressed by GV1001 treatment, which decreased the activation of pancreatic stellate cells and reduced fibrosis. Thus, it seems that the increased production of pro-inflammatory cytokines such as TNF-α, IL-1β, and IL-6 in gemcitabine-treated PDAC xenograft mice was efficiently suppressed by GV1001 treatment, which decreased the activation of pancreatic stellate cells and consequently reduced fibrosis.

We could find out GV1001 decreased the levels of TNF-α, IL-1β, and IL-6 as well as the population of activated pancreatic stellate cells. Thus, it seems that the increased production of pro-inflammatory cytokines such as TNF-α, IL-1β, and IL-6 in gemcitabine-treated PDAC xenograft mice was efficiently suppressed by GV1001 treatment, which decreased the activation of pancreatic stellate cells and consequently reduced fibrosis. The potential sources of these cytokines would be pancreatic epithelial cells, pancreatic cancer cells, pancreatic stellate cells and innate immune cells like macrophages [52, 53]. Especially, it was reported pancreatic acinar cells expressing TNF-α, IL-1 and IL-6 activate PSCs [54–56]. Furthermore, tumor-infiltrating macrophages were known to be related with ECM remodeling and cancer fibrosis [57]. Recently, it was reported that CD40 activation on macrophages released interferon-γ and CCL2 and induced MMP-dependent fibrosis degradation in pancreatic carcinoma, resulting in the enhanced chemotherapy efficacy [39, 58]. Therefore, it should be more investigated the target cells of GV1001 and its related mechanism.

Here, we report GV1001 did not have direct anti-cancer effects on PDACs, however, GV1001 combined with gemcitabine treatment showed significant loss of fibrosis in tumor tissue as well as tumor cell death and it might be the key component to understand the possible synergistic effects of anti-cancer drug delivery in PDAC treatment. Here, we would like to address that GV1001 might have robust effects in enhancing drug delivery and become a promising tool to overcome chemo-resistance in a treatment of PDACs.

## MATERIALS AND METHODS

### Pancreatic ductal adenocarcinoma cells (PDAC cells)

Human pancreatic cancer cell lines, PANC1 and AsPC1 were obtained from Korea Cell Line Bank and maintained in RPMI1640 medium (WELGENE, Daegu, Korea) containing 10% heat-inactivated fetal bovine serum (Life Technologies, Grand Island, NY, USA) and antibiotics (100 U/ml of penicillin and 100 μg/ml streptomycin; Life Technologies). They were incubated at 37°C and 5% CO_2_. AsPC1 cells were cultured, and stained with anti-CD133 antibody (Miltenyi Biotec, Bergisch Gladbach, Germany) for 15 min on ice with rotation. Followed by washing with a buffer (phosphate buffered saline (PBS) containing 1% bovine serum albumin (BSA) and 0.01% sodium azide) twice, CD133+ AsPC1 cells were isolated by FACSAria (BD Bioscience). Isolated CD133+ AsPC1 cells were incubated in CO_2_ incubator, and subcultured for tumor xenograft.

### Apoptosis determination

PANC1 and AsPC1 were cultured and divided into 3 groups according to the treatment: (i) Control, (ii) GV1001 (20 μM), (iii) GV1001 (40 μM). After treatments for 24 hrs, PDAC cells were washed with cold PBS and then resuspended in 1× Annexin V binding buffer (BD Biosciences, San Jose, CA, USA) at a concentration of 1×10^6^ cells/ml. After PDAC cells (1×10^5^ cells/tube) were incubated with Annexin V-fluorescein isothiocyanate (FITC) for 15 minutes on ice in the dark, 7-Amino-Actinomycin (7-AAD, BD Biosciences) was added to tube containing cells before analysis. The degree of apoptosis was analyzed by flow cytometry, and the proportion of stained cells in each quadrant was quantified with CellQuest software (BD Biosciences).

### Cell proliferation assay

AsPC1 cells were plated into 96-well plates at a density of 2×10^4^ cells/well and cultured in the presence or absence of GV1001 in various concentrations from 5 μM to 100 μM for 24 hrs. Cell proliferation was then measured with the Cell Counting Kit-8 (CCK-8) assay (Dojindo, Kumamoto, Japan) according to the manufacturer's instruction. Absorbance values were measured using the microplate reader and SoftmaxPro software (Molecular Devices, Sunnyvale, CA, USA).

### Animals

Seven-week-old male BALB/c nude mice were purchased from Chung-Ang Laboratory Animal (Seoul, Korea). Mice were housed under specific pathogen-free conditions, and a γ-ray-irradiated laboratory rodent diet (Purina Korea, Gyeonggi-do, Korea) and autoclaved water were provided *ad libitum*. All the protocols for the animal experiments were reviewed and approved by the Institutional Animal Care and Use Committee at Seoul National University Hospital (#13-0717) and Seoul National University (#131008-3). All animal procedures were in consistent with the “Guide for the Care and Use of Laboratory Animals” issued by the Institute of Laboratory Animal Resources Commission on Life Science, US National Research Council.

### Establishment of PDAC xenograft tumor model and treatment

To generate tumors, human PDAC cells (PANC1, AsPC1, CD133+ AsPC1, 1 × 10^6^ cells/50 μl PBS) were subcutaneously inoculated with 50 μl of Matrigel in both sides of buttocks. All mice were divided into 4 groups randomly with 5 mice in each group. The treatment was began after 10 days from the PDAC cell injection with confirmation of gross xenograft tumor in each mouse: (i) control (vehicle alone), (ii) gemcitabine (twice-a-week intraperitoneal injection at 125 mg/kg for 2 weeks), (iii) GV1001 (every day subcutaneous injection at 50 μg/ea for 2 weeks), (iv) gemcitabine (twice-a-week intraperitoneal injection of at 125 mg/kg for 2 weeks) and GV1001 (every day subcutaneous injection at 50 μg/ea for 2 weeks). The body weight and the tumor size of each mouse were measured using electronic scale and caliper. Tumor volume was calculated by the following formula: tumor volume = (length x width^2^) x π/6 [34].

### Blood sample and cytokine measurement

Blood samples were collected from the infra-orbital venous plexus with heparinized capillary at the time of sacrifice, and centrifuged with 14000 rpm for 30 min at 4°C. Quantikine human enzyme-linked immunosorbent assay (ELISA) kits for interleukin (IL)-6, IL-1β and tumor necrosis factor TNF-α were purchased from R&D systems (Minneapolis, MN, USA). Analyses were performed according to the manufacture's protocol for each ELISA kit, assayed in triplicate, and read on a Molecular Devices microplate reader at 450 nm (Menlo Park, CA, USA).

### Harvest and histological examination of the xenograft tumor tissue

The mice were immediately sacrificed after the 2 weeks of treatment protocol. Tumors were excised, fixed with 4 % paraformaldehyde (PFA) at 4°C and processed for paraffin embedding. Tissue sections (5 μm thickness) were prepared using a microtome, and placed on glass slides. Serial sections were cut from paraffin-embedded tumor tissues, and stained with Hematoxylin & Eosin (H&E) and Masson's trichrome (Sigma, St. Louis, MO, USA) according to the manufacturer's instruction. Masson's trichrome–stained sections were evaluated to quantify the amount of fibrosis calculating the area occupied by blue-stained collagen using ImageJ program. Otherwise, tissue sections were deparaffinized with xylene and hydrated with alcohol series. Then, antigenic retrieval was performed by heating with 0.1 M citrate buffer (pH 6.0) under microwave after hydration. Followed by blocking endogenous peroxidase with H_2_O_2_ and inhibiting nonspecific signals with 5 % goat serum, sections were incubated with primary antibody against alpha-smooth muscle actin (α-SMA, 1:100, Sigma) at 4°C overnight in a humidified chamber. Then, sections were incubated with a biotinylated anti-rabbit antibody (Vector laboratories, Burlingame, CA, USA) for 1 hr at room temperature. And then ABC solution (Vector laboratories) was loaded on sections for 30 min. DAB kit (Vector laboratories) was used for chromogenic detection. Subsequent to dehydration and clearing, the sections were mounted with DPX mountant (Fluka, St. Louis, MO, USA) and observed with light microscope (Olympus, Center Valley, PA, USA). The number of anti-alpha-SMA (α-SMA) immunoreactive stellate cells was evaluated by pathologist. Ten random fields of high-magnification (x400) of light microscope were searched to detect α-SMA immunoreactive fibroblast in each case.

### Statistical analysis

All experimental results represent at least 3 independent experiments using cells from a minimum of three separate isolations. Results for continuous variables are expressed as means ± standard error of mean (SEM) and compared with the Kruskal-Wallis one-way analysis of variance (ANOVA) followed by Dunn's multiple comparison test or Newman-Keuls multiple comparison test. *P* < 0.05 was considered statistically significant. Analysis was performed with GraphPad Prism version 5.04 (GraphPad Software Inc., La Jolla, CA).

## SUPPLEMENTARY FIGURES


