# Supplementary material for: The anti-fibrotic effect of GV1001 combined with gemcitabine on treatment of pancreatic ductal adenocarcinoma
Source: Oncotarget. 2016 Sep 16;7(46):75081–93. doi: 10.18632/oncotarget.12057 (PMC5342724; doi:10.18632/oncotarget.12057)
Supplement: Supplementary file 1 [file oncotarget-07-75081-s001.pdf]

The anti-fibrotic effect of GV1001 combined with gemcitabine on treatment of pancreatic ductal adenocarcinoma

SUPPLEMENTARY FIGURES

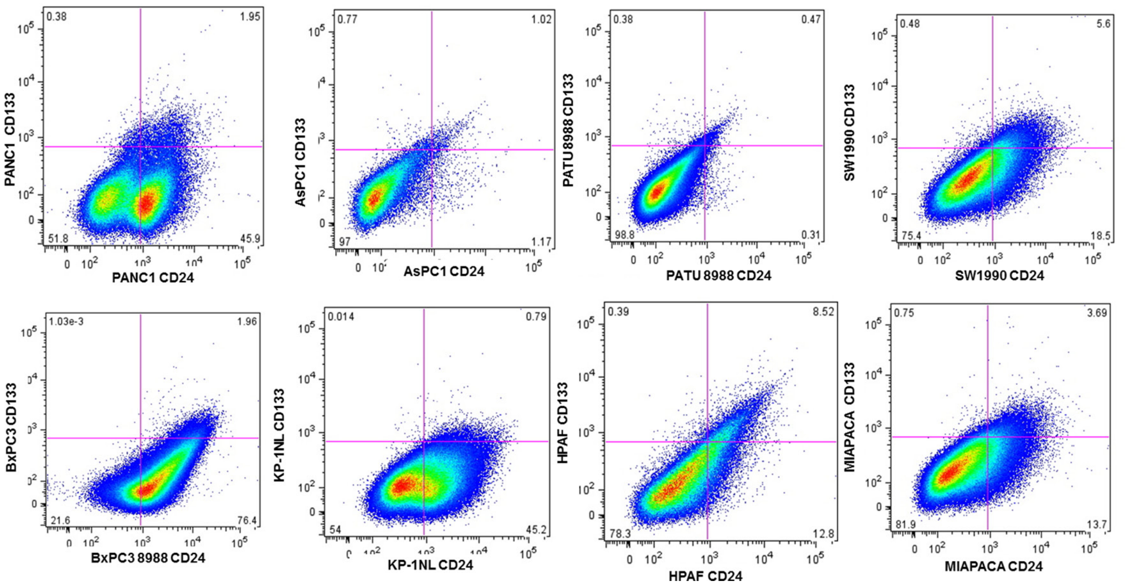

Supplementary Figure S1: FACS analysis of CD133+ PDAC cell lines.

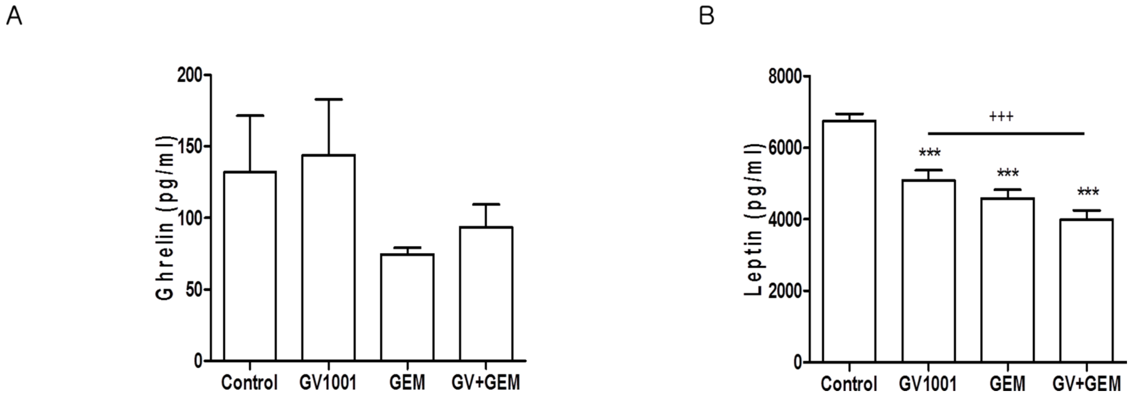

Supplementary Figure S2: Ghrelin and Leptin level among the different treatment groups. A. Ghrelin level was measured among the different treatment group **B.** Leptin level was measured among the different treatment group.
